# Enhanced Removal of Copper Ions from Aqueous Solution by Citrate-Stabilized Amorphous Calcium Phosphate Nanoparticles/Sodium Alginate Composite Hydrogel Beads

**DOI:** 10.3390/nano16110662

**Published:** 2026-05-24

**Authors:** Miaomiao Wang, Yuwei Jiang, Junjun Tan

**Affiliations:** Hubei Provincial Key Laboratory of Green Materials for Light Industry, School of Material Science and Chemical Engineering, Hubei University of Technology, Wuhan 430068, China

**Keywords:** sodium citrate, sodium alginate, amorphous calcium phosphate nanoparticle, copper removal, gel beads

## Abstract

Although amorphous calcium phosphate (ACP) has been extensively employed as a biomaterial in dental and orthopedic fields, its exploration for environmental applications—particularly in potentially toxic element remediation—remains notably limited in the scientific literature. This study reports the rational design of a multifunctional adsorbent by integrating sodium citrate-stabilized ACP (Cit-ACP) nanoparticles into calcium-crosslinked sodium alginate (SA) hydrogel beads for selective Cu^2+^ sequestration from aqueous systems. Comprehensive sorption assessments revealed that equilibrium uptake aligned with the Freundlich isotherm (indicating heterogeneous surface interactions), while kinetic profiles adhered to pseudo-second-order behavior, characteristic of chemisorption-driven processes. Under optimized operational parameters (pH 5.0, 45 °C), the Cit-ACP/SA composite attained an exceptional maximum adsorption amount of 307.76 mg/g. Thermodynamic analysis further confirmed the spontaneity (ΔG° < 0) and endothermic nature (ΔH° > 0) of the process. Multi-technique characterization (XPS, FTIR, XRD, pH trajectory) elucidated a dual-mode adsorption mechanism: (i) ion exchange between aqueous Cu^2+^ and structural Ca^2+^ within both the alginate matrix and ACP framework; and (ii) in situ surface precipitation yielding copper-substituted hydroxyapatite. Owing to its facile aqueous-phase synthesis, superior adsorption performance, biodegradability, macroscopic bead morphology enabling rapid separation, and robust selectivity in complex matrices, the Cit-ACP/SA composite presents a sustainable, scalable, and eco-compatible platform for practical remediation of copper-contaminated wastewater.

## 1. Introduction

As a requisite micronutrient for humans and animals, copper underpins core physiological operations—most notably enzymatic activity and iron metabolic regulation. [1]. However, chronic exposure to copper-contaminated drinking water exceeding the WHO guideline value (2.0 mg/L) correlates with multisystem toxicity, clinically manifesting as hepatic and renal impairment, gastrointestinal disturbances, and neurological dysfunction [2].

Owing to the significant health and ecological risks posed by elevated copper concentrations in aquatic systems, diverse remediation strategies have been investigated [3,4,5], encompassing chemical precipitation [6], ion exchange [7], membrane filtration [8], electrocoagulation [9], and adsorption [10,11]. Within this spectrum, adsorption has emerged as a particularly advantageous technique for potentially toxic element ion sequestration, distinguished by its operational efficiency, environmental sustainability, and economic feasibility [4,5]. Extensive research has evaluated numerous sorbent materials for copper capture—including hydroxyapatite [12], metal–organic frameworks (MOFs) [13], clay minerals [14], composite hydrogels [15], and biochar [16,17]. Nonetheless, the broader implementation of conventional sorbents remains constrained by persistent challenges: moderate binding capacities, limited target specificity, kinetically sluggish uptake, and regeneration protocols requiring substantial energy or chemical input.

As a transient metastable phase within the calcium phosphate family, amorphous calcium phosphate (ACP) commonly emerges during the nascent stages of biomineralization [18,19], with a hydrated composition typically denoted as [Ca_3_(PO_4_)_2_]n·zH_2_O. Its favorable biological profile—characterized by pronounced bioactivity, high biocompatibility, and minimal cytotoxicity—has spurred extensive investigation into its utility across diverse biomedical domains, including bone regeneration, drug delivery, and dental therapeutics [20,21,22,23,24].

However, pristine amorphous calcium phosphate (ACP) exhibits inherent thermodynamic instability under ambient conditions, readily undergoing crystallization into hydroxyapatite (HAp)—a phase transition that critically limits its functional utility [25]. To suppress this transformation, incorporation of molecular stabilizers such as sodium citrate, polyethylene glycol (PEG), or casein has been widely adopted [21,26,27]. Among these, sodium citrate demonstrates superior efficacy: the resultant citrate-stabilized ACP (Cit-ACP) achieves a markedly enhanced specific surface area and maintains structural fidelity as a dry powder at room temperature for up to four years [25]. Critically, the dense presentation of multifunctional surface moieties—including phosphate, carboxylate, and hydroxyl groups—endows Cit-ACP nanoparticles with robust chelating capacity, positioning them as highly promising adsorbents for potentially toxic element ion sequestration.

To overcome the limitations of standalone Cit-ACP in aqueous remediation—particularly regarding operational handling and recyclability—its integration into a biopolymer scaffold offers a synergistic solution. Sodium alginate (SA), a naturally occurring polysaccharide, forms mechanically robust hydrogel beads through ionic crosslinking with Ca^2+^ ions. These beads are widely adopted as adsorbent matrices due to their tunable porosity, operational simplicity, and biodegradability [28,29]. Embedding Cit-ACP nanoparticles within the SA network not only amplifies copper-binding capacity through complementary active sites but also enables rapid solid–liquid separation without centrifugation or filtration.

Although alginate-based adsorbents and calcium phosphate materials have been individually explored for potentially toxic element removal, the combination of citrate-stabilized amorphous calcium phosphate with alginate for Cu(II) adsorption has not been reported. Unlike conventional crystalline hydroxyapatite or simple alginate beads, the metastable amorphous phosphate phase may offer a high density of undercoordinated phosphate sites, while citrate acts as both a stabilizer and an additional metal-binding ligand. This unique composition is expected to provide enhanced adsorption performance through a synergistic mechanism. This study presents the first systematic investigation of Cu^2+^ sequestration using Cit-ACP/SA hydrogel beads synthesized via CaCl_2_-mediated crosslinking. We comprehensively evaluate adsorption performance through capacity quantification, kinetic modeling, and isotherm analysis, while probing the influence of critical parameters including solution pH, initial metal concentration, and competing ions. Complementary physicochemical characterizations further decipher the molecular-scale mechanisms governing Cu^2+^ capture, providing foundational insights for designing advanced biopolymer-based adsorbents in potentially toxic element remediation.

## 2. Materials and Methods

### 2.1. Materials

The following reagents were used in this study. Calcium chloride dihydrate (CaCl_2_·2H_2_O, ≥99.0%), trisodium citrate dihydrate (C_6_H_5_Na_3_O_7_·2H_2_O, ≥99.0%), trisodium phosphate dodecahydrate (Na_3_PO_4_·12H_2_O, ≥98.0%), sodium alginate ((C_6_H_7_NaO_6_)_n_, chemically pure), copper(II) nitrate trihydrate (Cu(NO_3_)_2_·3H_2_O, ≥99.99%), and sodium hydroxide (NaOH, ≥97.0%) were supplied by Shanghai Aladdin Bio-Chem Technology Co., Ltd. (Shanghai, China). Nitric acid (HNO_3_, ≥68%), magnesium chloride hexahydrate (MgCl_2_·6H_2_O, ≥99.0%), sodium chloride (NaCl, ≥99.0%), and potassium chloride (KCl, ≥99.0%) came from Sinopharm Chemical Reagent Co., Ltd. (Shanghai, China). Sodium diethyldithiocarbamate trihydrate (C_5_H_10_NNaS_2_·3H_2_O, ≥99%) was purchased from Macklin Inc. (Shanghai, China). All chemicals were employed directly without additional purification, and deionized water was used throughout all experiments.

### 2.2. Synthesis of Cit-ACP Nanoparticles

To fabricate Cit-ACP nanoparticles, 40 g of deionized water containing 0.05 mol of sodium citrate was dropwise added over 30 min to a continuously agitated calcium chloride solution (0.05 mol dissolved in 40 g of water). Subsequently, under vigorous stirring for one hour, a sodium phosphate solution (0.03 mol in 50 g of water) was slowly introduced into the mixture. The resulting precipitate was purified via a two-step centrifugation and rinsed with deionized water. After purification, the precipitate was re-dispersed in water. For the final composite preparation, a suspension of 1 g of sodium alginate in 50 g of water was combined with the purified Cit-ACP nanoparticle dispersion.

### 2.3. Fabrication of Cit-ACP/SA Gel Beads

To obtain a homogeneous dispersion, 1.5 g of purified Cit-ACP nanoparticles were dispersed in 50 g of deionized water inside a 250 mL beaker under magnetic agitation for 5 min. Subsequently, a 2 wt% sodium alginate solution (prepared by dissolving 1 g SA in 50 g water) was added dropwise using a peristaltic pump set at 4.5 rpm, followed by another 5 min of stirring to guarantee even mixing. The resulting mixture was then dripped into a 2 wt% CaCl_2_ crosslinking bath, where spherical beads formed via ionic gelation over a period of 2 h. This composite, containing 1.5 g of Cit-ACP, was labeled Cit-ACP/SA-4 and used as the main adsorbent. To systematically evaluate the influence of Cit-ACP loading on adsorptive performance, a series of composites were fabricated with different Cit-ACP contents. The preparation procedures for the composites were closely similar to those for sample Cit-ACP/SA-4, except for the added mass of Cit-ACP. The detailed sample codes and the corresponding mass ratios of SA to Cit-ACP are listed in Table 1.

### 2.4. Characterization

Physicochemical characterization was performed using an integrated suite of techniques. XRD patterns were collected on a Bruker D8 Advance diffractometer (Bruker AXS, Karlsruhe, Germany) equipped with Cu Kα radiation (λ = 0.15406 nm), over a 2θ range of 5–80° at a scanning speed of 6°·min^−1^. Morphological features were visualized by FE-SEM (Hitachi SU-8010, Tokyo, Japan) following a brief gold sputtering step (30 s, MSP-2S coater) to ensure electrical conductivity. XPS analysis (Thermo Scientific ESCALAB 250Xi, Al Kα source, Waltham, MA, USA) was conducted to determine surface elemental states. N_2_ adsorption–desorption isotherms recorded at 77 K (Micromeritics 3Flex, Norcross, GA, USA) provided textural properties: the BET method yielded specific surface areas, and the BJH model applied to the desorption branch gave pore size distributions. ATR-FTIR spectroscopy (Nicolet iS5, wavenumber range 400–4000 cm^−1^, Waltham, MA, USA) identified functional groups. All measurements were conducted under reproducible conditions to allow cross-technique comparison and data reliability.

### 2.5. Batch Adsorption Assays

To determine the Cu(II) removal capacity, a series of batch experiments were carried out. In each test, 20 mg of adsorbent was added to 25 mL of Cu(II) solution (initial concentration ranging from 5 to 300 mg/L, with pH adjusted to 5.0) placed in centrifuge tubes. The mixtures were shaken at 100 rpm for 24 h at 25 °C to reach equilibrium. After that, the samples were centrifuged at 8000 rpm for 10 min. The remaining Cu(II) concentration in the supernatant was then measured using a spectrophotometer (Shimadzu UV-Mini 1280, Kyoto, Japan). In brief, the Cu(II) concentration of solution was measured spectrophotometrically after complexation with sodium diethyldithiocarbamate (DDTC). The yellow–brown Cu(II)–DDTC complex was formed in ammoniacal buffer, and its absorbance was measured at 452 nm against a reagent blank. The concentration was determined from a calibration curve prepared with standard Cu(II) solutions, as shown in Figure S1 (see Supporting Information).

The equilibrium adsorption amount ($q_{e}$, mg/g) was calculated using Equation (1) [30]

(1) $$q_{e}=\frac{(C_{0}-C_{e})V}{m}$$

where $C_{0}$ and $C_{e}$ denote initial and equilibrium Cu(II) concentrations (mg/L), V is solution volume (L), and m represents adsorbent mass (g). All experiments were conducted in triplicate under strictly controlled thermal conditions (25 ± 0.5 °C) to ensure reproducibility.

Kinetic Studies: The time-dependent behavior of Cu^2+^ uptake was assessed at an adsorbent loading of 0.8 g/L, initial Cu^2+^ concentration of 100 mg/L, pH 5.0, and a constant temperature of 25 °C. The Cu^2+^ solution was dispensed into centrifuge tubes, followed immediately by the addition of the adsorbent. The suspensions were shaken at 100 rpm in a temperature-controlled orbital shaker, and samples were taken at preset intervals. After centrifugation (8000 rpm, 10 min), the supernatants were analyzed spectrophotometrically for residual metal ions. The experimental data were then fit to the pseudo-first-order (Equation (2)) and pseudo-second-order (Equation (3)) kinetic models to determine the rate-limiting step [15]:

Pseudo-first-order:

(2) $$\ln\left(q_{e}\right.-q_{t})=lnq_{e}-k_{1}t$$

Pseudo-second-order:

(3) $$\frac{t}{q_{t}}=\frac{1}{K_{2}q_{e}^{2}}+\frac{t}{q_{t}}$$

Here, $q_{t}$ (mg/g) and $q_{e}$ (mg/g) denote the adsorption amounts at time t (min) and at equilibrium, respectively; $k_{1}$ (min^−1^) is the pseudo-first-order rate constant, and $k_{2}$ (g·mg^−1^·min^−1^) is the pseudo-second-order rate constant. Linear regression was employed to determine the optimized kinetic parameters, improving model robustness and aiding mechanistic understanding.

Isotherm Studies: Equilibrium uptake of Cu^2+^ was investigated at initial concentrations varying from 5 to 300 mg/L, with an adsorbent loading of 0.8 g/L (20 mg in 25 mL), pH maintained at 5.0, and a contact period of 24 h to ensure full equilibration. Three temperature levels (25, 35, and 45 °C) were tested in a thermostatic shaker at 100 rpm. Following centrifugation (8000 rpm, 10 min) for phase separation, the residual Cu^2+^ concentrations were determined spectrophotometrically. Linearized versions of the Langmuir (Equation (4)) and Freundlich (Equation (5)) isotherms were used to fit the equilibrium data [30]:

Langmuir model:

(4) $$\frac{C_{e}}{q_{e}}=\frac{1}{K_{L}q_{m}}+\frac{C_{e}}{q_{m}}$$

Freundlich empirical equation:

(5) $$\ln q_{e}=\ln K_{F}+\frac{1}{n}\ln C_{e}$$

Here, the equilibrium adsorption amount and the Cu^2+^ concentration is denoted by $q_{e}$ (mg/g) and $C_{e}$ (mg/L), respectively. $q_{m}$ (mg/g) corresponds to the theoretical monolayer saturation capacity. $K_{L}$ (L/mg) is a measure of adsorption affinity. $K_{F}$ ((mg/g) (L/mg)^1/*n*^) represents the uptake under unit concentration, and the exponent 1/n describes surface heterogeneity and adsorption strength (values < 1 being indicative of favorable adsorption). Linear regression analysis was applied to determine the model parameters, and the coefficient of determination ($R^{2}$) was employed to evaluate model suitability. All measurements were performed in triplicate to guarantee both statistical reliability and reproducibility of the thermodynamic conclusions.

pH Effect: The dependence of Cu^2+^ adsorption on the initial pH was investigated across the 3.0–5.5 interval. Solution pH values were accurately adjusted beforehand using 0.1 M HCl or NaOH. Constant experimental parameters were maintained throughout: adsorbent dose = 0.8 g/L, initial Cu^2+^ = 200 mg/L, temperature = 25 °C, and contact time = 24 h. The chosen pH window prevented copper hydrolysis while enabling the evaluation of protonation effects on the surface binding sites.

Coexisting Ions Effect: To investigate the selectivity of the adsorbent under realistic water conditions, we examined the influence of common cations (Na^+^, K^+^, Mg^2+^, Ca^2+^) on Cu^2+^ removal. Each competing ion was added at varying concentrations (0.1, 0.3, 0.6, and 1.2 mM) to solutions containing 50 mg/L Cu^2+^. The tests were run under fixed parameters (adsorbent dose: 0.8 g/L; temperature: 25 °C; contact time: 24 h). The adsorption efficiency was then calculated by comparison with ion-free control samples. This experimental setup allowed a quantitative assessment of ionic competition intensity and the material’s practical applicability in complex aqueous matrices.

## 3. Results and Discussion

### 3.1. Characterization of SA/ACP Gel Beads

**Figure 1:**
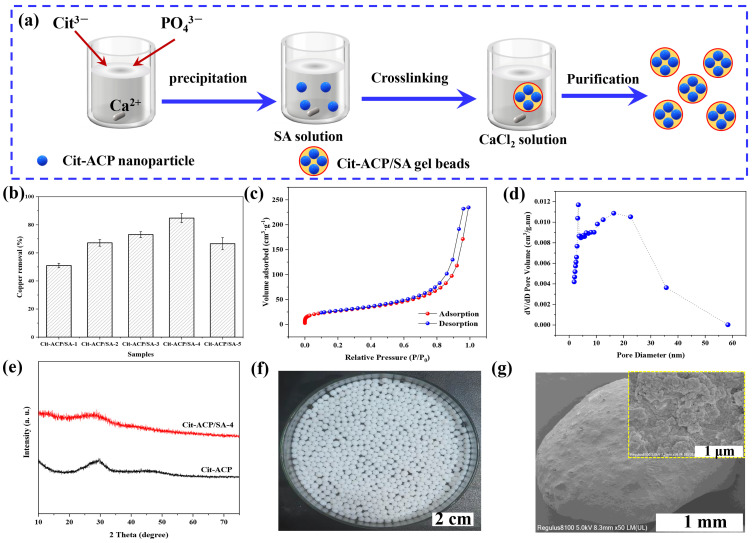
(**a**) Schematic illustration of sample preparation; (**b**) copper removal efficiency of different samples; (**c**) BET surface area, (**d**) pore distribution, (**e**) XRD pattern, (**f**) digital image, and (**g**) SEM image of the sample Cit-ACP/SA-4.

Figure 1a schematically illustrates the preparation process of Cit-ACP/SA gel beads: (1) calcium ions, phosphate ions, and citrate ions were mixed at 25 °C for 1 h to form Cit-ACP nanoparticles; (2) the resulting product was mixed with a sodium alginate solution at 25 °C for 1 h to form a dispersion; (3) the dispersion was dropped into a CaCl_2_ solution to obtain gel beads; and (4) the beads were purified by centrifugation to yield the final Cit-ACP/SA composite beads.

As illustrated in Figure 1b, copper removal efficiency exhibits a pronounced dependence on Cit-ACP loading within the composite beads. The pure sodium alginate matrix (Cit-ACP/SA-1) achieved a baseline removal of 50.98 ± 1.6%. Incremental Cit-ACP incorporation (0.25–1.5 g) progressively enhanced performance, with efficiencies rising from 67.14 ± 2.4% to a maximum of 84.77 ± 3.2% at the optimal loading (Cit-ACP/SA-4). Notably, exceeding this threshold (4.0 g, Cit-ACP/SA-5) triggered a decline to 66.55 ± 4.3%, suggesting structural or kinetic limitations at elevated nanoparticle concentrations. Critically, Cit-ACP/SA-4 demonstrated a 33.79 ± 2.4% higher removal efficiency relative to the unmodified SA control, unequivocally validating the synergistic role of citrate-stabilized amorphous calcium phosphate in amplifying the composite’s copper sequestration capability. To evaluate the effect of adsorbent dosage on Cu(II) removal, the Cit-ACP/SA-4 beads were added to a 200 mg/L Cu(II) solution at dosages ranging from 0.2 to 1.0 g/L (Figure S2 in Supporting Information). As the adsorbent dosage increased, the removal efficiency monotonically increased from 35.40 ± 2.8% to 95.06 ± 3.7%, while the equilibrium adsorption amount decreased from approximately 354.02 ± 8.7 mg/g to 190.13 ± 9.5 mg/g. Considering both removal efficiency and adsorption capacity, an adsorbent dosage of 0.8 g/L was selected for all subsequent adsorption experiments.

Textural and structural properties of the Cit-ACP/SA-4 composite were systematically characterized. Nitrogen physisorption analysis (Figure 1c) revealed a type IVa isotherm with H3-type hysteresis per IUPAC criteria, confirming a well-defined mesoporous framework. The material exhibited a specific surface area of 90.15 m^2^/g. Figure 1d shows the pore size distribution of the Cit-ACP/SA-4 sample. The pore diameters range from 2 to 60 nm, with a mean pore size of 15.27 nm—features that are conducive to enhanced mass transport and adsorption accessibility.

XRD patterns (Figure 1e) further validate structural integrity. Both Cit-ACP and Cit-ACP/SA-4 displayed broad amorphous halos centered near 2θ = 30° without discernible crystalline reflections, confirming the absence of hydroxyapatite or other crystalline calcium phosphate phases. This demonstrates successful preservation of ACP’s amorphous state throughout alginate encapsulation and crosslinking.

Morphological evaluation (Figure 1f,g) showed uniformly spherical beads (mean diameter: 2.5 mm) with macroscopically smooth surfaces. High-resolution SEM imaging revealed a microscopically rough, hierarchically porous exterior. This dual-scale architecture—combining macroscopic bead uniformity with nanoscale surface porosity—creates an extensive reactive interface that facilitates rapid diffusion and efficient binding of Cu^2+^ ions, directly contributing to the composite’s superior adsorption performance.

### 3.2. Cu(II) Adsorption Characteristics of the Cit-ACP/SA-4 Beads

#### 3.2.1. Kinetic Behavior of Cu(II) Uptake

**Figure 2:**
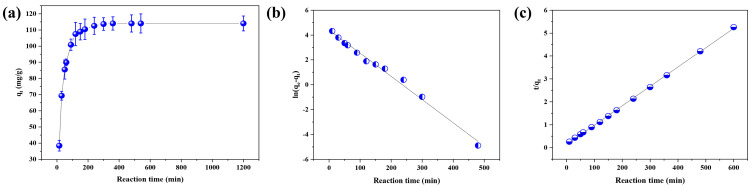
Kinetic profiling of Cu^2+^ uptake by Cit-ACP/SA-4 composite beads: (**a**) time-dependent adsorption amount evolution; (**b**) linearized pseudo-first-order kinetic fit; (**c**) linearized pseudo-second-order kinetic fit.

Figure 2a delineates the time-resolved adsorption behavior of Cu^2+^ on Cit-ACP/SA-4 composite beads, revealing an initial rapid uptake phase (0–120 min) followed by progressive saturation, with equilibrium attained at approximately 240 min. To discern the rate-controlling mechanism, kinetic datasets were subjected to linear regression against pseudo-first-order and pseudo-second-order models (Figure 2b,c; Table 2). The pseudo-second-order model exhibited markedly superior fidelity to the experimental trajectory, substantiated by two decisive metrics: (i) the theoretically derived equilibrium amount ($q_{e,\mathrm{cal}}=118.3$ mg/g) aligned closely with the measured value ($q_{e,\exp}=114.05$ mg/g), whereas the pseudo-first-order prediction (91.8 mg/g) showed significant deviation; (ii) an exceptionally high coefficient of determination ($R^{2}=0.999$) versus $R^{2}=0.991$ for pseudo-first-order. These quantitative indicators collectively confirm that Cu^2+^ adsorption onto Cit-ACP/SA-4 adheres to pseudo-second-order kinetics, implying that chemisorption—governed by valence forces through electron sharing or exchange—predominates the rate-determining step.

#### 3.2.2. Effect of Initial Performance of the Sample SA/ACP-4

**Figure 3:**
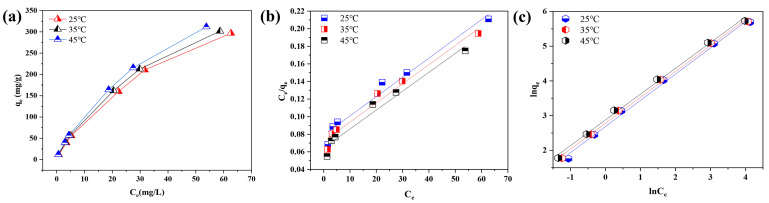
(**a**) Equilibrium uptake profiles of Cu^2+^ on Cit-ACP/SA-4 composite beads; (**b**) Langmuir linearized representation; (**c**) Freundlich linearized representation.

Equilibrium adsorption capacities of the Cit-ACP/SA-4 composite were quantified across a gradient of initial Cu^2+^ concentrations (Figure 3a). Experimental isotherm data were rigorously fitted to Langmuir and Freundlich models [31], with linearized representations presented in Figure 3b,c and corresponding parameters summarized in Table 3. The Freundlich model yielded significantly higher correlation coefficients ($R^{2}$), indicating that Cu^2+^ sorption occurs on a surface characterized by energetically heterogeneous binding sites—a behavior inconsistent with the homogeneous monolayer assumption inherent to the Langmuir framework. Concurrently, equilibrium uptake amount exhibited positive dependencies on both solution temperature and initial Cu^2+^ concentration, reflecting enhanced thermodynamic driving force and greater availability of active sites under elevated concentration and thermal conditions. This dual dependence further supports the chemisorption-dominated mechanism inferred from kinetic analysis.

#### 3.2.3. Adsorption Thermodynamic Parameters Analysis

Thermodynamic parameters—standard Gibbs free energy change (ΔG°), enthalpy change (ΔH°), and entropy change (ΔS°)—were evaluated to elucidate the energetics, spontaneity, and temperature dependence of Cu^2+^ sorption onto the Cit-ACP/SA-4 composite. Parameters were derived from equilibrium isotherm data acquired at 25, 35, and 45 °C [15,32].

**Figure 4:**
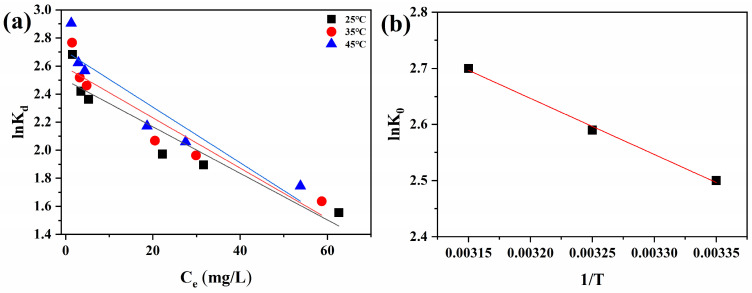
Thermodynamic analysis of Cu^2+^ sorption on Cit-ACP/SA-4 composite beads: (**a**) dependence of ln *K*_d_ on equilibrium concentration (*C*_e_); (**b**) Van’t Hoff plot of ln *K*_0_ versus reciprocal absolute temperature (1/*T*).

The thermodynamic equilibrium constant (K°, L·g^−1^) was obtained by extrapolating plots of ln *K*_d_ versus *C*_e_ (*K*_d_ = q_e_/C_e_) to *C*_e_ → 0 (Figure 4a), where the *y*-intercept corresponds to ln *K*°. Subsequently, ΔH° and ΔS° were determined from the slope and intercept of the Van’t Hoff plot (ln *K*° versus 1/*T*; Figure 4b), consistent with:

ln K° = −ΔH°/(RT) + ΔS°/R(6)

ΔG° was then computed via:

ΔG° = ΔH − TΔS°(7)

(T: absolute temperature in K; R: 8.314 J·mol^−1^·K^−1^).

As summarized in Table 4, the positive Δ*H*° value (+8.314 kJ·mol^−1^) confirms the endothermic nature of adsorption, implying that thermal energy facilitates Cu^2+^ binding—likely through enhanced diffusion or activation of surface sites. The consistently negative Δ*G*° values (−6.18 to −7.15 kJ·mol^−1^) across all temperatures indicate that the equilibrium favors the adsorption of Cu(II) onto the Cit ACP/SA beads under standard conditions, with increasing negativity at elevated temperatures indicating strengthened thermodynamic driving force. The positive Δ*S*° (+48.64 J·mol^−1^·K^−1^) further suggests increased randomness at the solid–liquid interface during adsorption, possibly due to displacement of hydrated water molecules from Cu^2+^ ions upon surface binding. Collectively, these findings align with the chemisorption mechanism inferred from kinetic and isotherm analyses, underscoring the temperature-enhanced efficacy of Cit-ACP/SA-4 for copper remediation.

#### 3.2.4. Influence of Solution pH and Competitive Cations on Cu^2+^ Uptake

**Figure 5:**
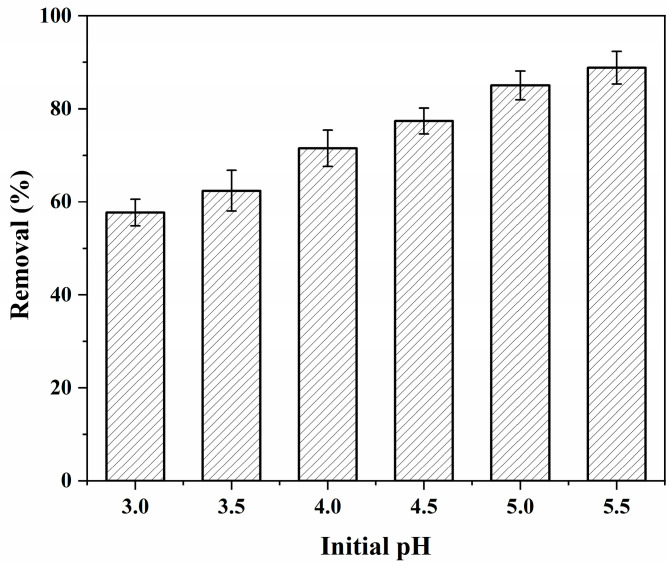
pH-dependent Cu^2+^ removal efficiency profile of Cit-ACP/SA-4 composite beads.

The removal efficiency of Cu^2+^ by the Cit-ACP/SA-4 composite is profoundly governed by solution chemistry. As delineated in Figure 5, Cu^2+^ sequestration exhibited a distinct pH-dependent profile across the operational window of 3.0–5.5: removal efficiency escalated from 57.69 ± 2.9% at pH 3.0 to 88.84 ± 3.5% at pH 5.5. This progressive enhancement correlates with reduced proton competition for surface binding sites and favorable deprotonation of functional groups (e.g., phosphate, carboxylate) at near-neutral conditions. Maximum performance was consistently achieved within pH 5.0–5.5, establishing this interval as the optimal operational range for copper remediation. Values beyond pH 5.5 were not examined to preclude potential Cu(OH)_2_ precipitation, ensuring observed effects reflect genuine adsorptive behavior rather than precipitation artifacts.

**Figure 6:**
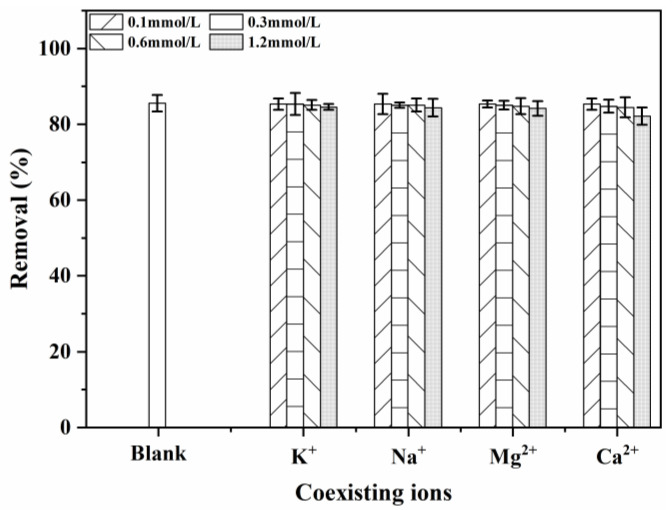
Influence of competitive cation on the Cu^2+^ removal efficiency of the Cit-ACP/SA-4 beads.

Figure 6 delineates the competitive interference of background electrolytes (Na^+^, K^+^, Mg^2+^, Ca^2+^) on Cu^2+^ sequestration by Cit-ACP/SA-4 composite beads. Monovalent cations (Na^+^, K^+^) and Mg^2+^ exerted negligible influence across the entire concentration gradient (0.1–1.2 mM), with adsorption efficiency deviating by <1% relative to the ion-free control. Conversely, Ca^2+^ induced a measurable, concentration-dependent suppression: Cu^2+^ removal declined progressively from 85.56 ± 2.2% (0 mM Ca^2+^) to 82.18 ± 2.3% (1.2 mM Ca^2+^), reflecting a 3.38 ± 4.1% absolute reduction. This selective attenuation arises from competitive coordination between Ca^2+^ and Cu^2+^ at phosphate- and carboxylate-functionalized binding sites.

#### 3.2.5. Adsorption Mechanism

To decipher the molecular-scale interactions governing Cu^2+^ sequestration by Cit-ACP/SA-4 composite beads, complementary surface-sensitive techniques (XPS, FTIR, XRD) were deployed on pristine and Cu^2+^-loaded specimens.

**Figure 7:**
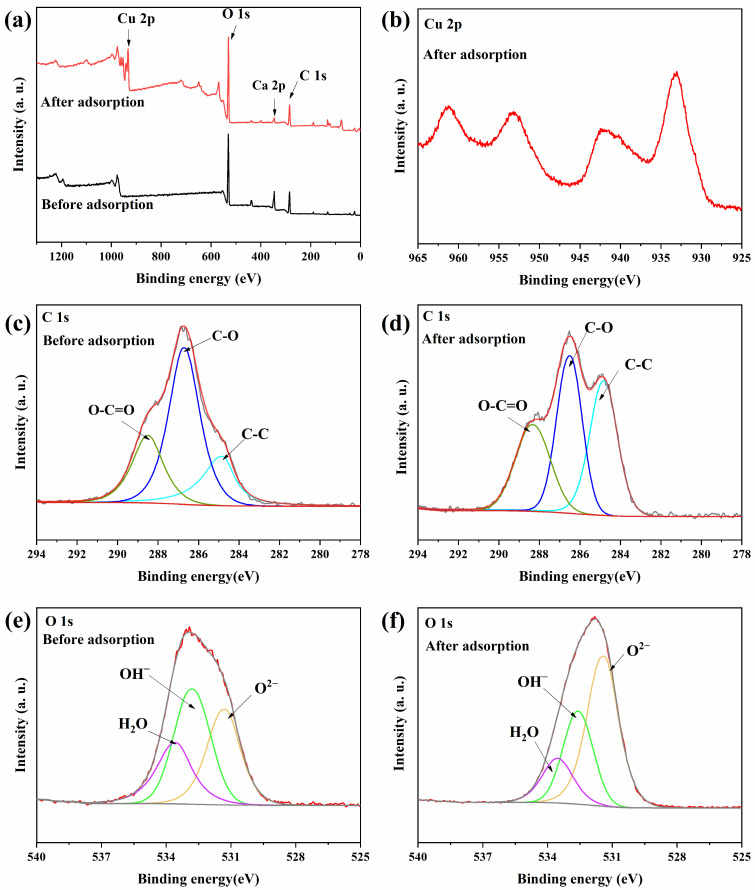
XPS characterization of Cit-ACP/SA-4 composite beads: (**a**) wide-scan spectra acquired prior to and following Cu^2+^ uptake; (**b**) high-resolution Cu 2p spectrum of the Cu^2+^ loaded composite; (**c**,**d**) deconvoluted C 1s core-level spectra in pristine versus Cu^2+^-exposed states; (**e**,**f**) corresponding deconvoluted O 1s spectra.

**Figure 8:**
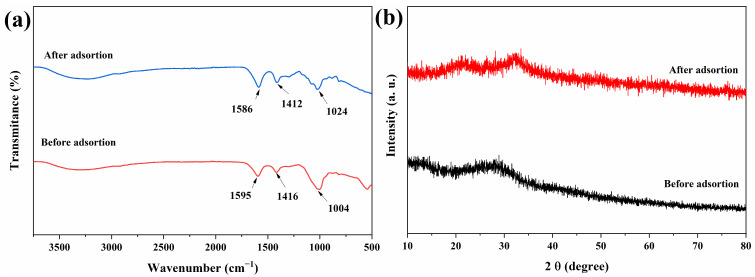
(**a**) FTIR of the sample Cit-ACP/SA-4 before and after adsorption; (**b**) XRD patterns of the sample Cit-ACP/SA-4 before and after adsorption.

XPS wide-scan spectra (Figure 7a) confirmed successful Cu^2+^ capture through emergent Cu 2p features (930–965 eV), corroborated by characteristic shake-up satellites in the high-resolution Cu 2p spectrum (Figure 8b) [33]. Concurrently, a marked attenuation of the Ca 2p signal (~345 eV) post-adsorption provides direct evidence of ion exchange between framework Ca^2+^ and aqueous Cu^2+^.

Deconvolution of C 1s spectra (Figure 7c,d; quantitative data in Table 5) revealed significant electronic reconfiguration upon Cu^2+^ binding [34]. The carboxylate component (O–C=O) increased from 19.7% to 27.6%, while hydroxyl-associated C–O decreased from 55.1% to 36.2%, and aliphatic C–C rose from 25.2% to 36.2%. This redistribution reflects preferential coordination of Cu^2+^ with carboxylate and hydroxyl moieties derived from sodium alginate and citrate stabilizers. Concurrent O 1s analysis (Figure 7e,f) further substantiated metal–ligand complexation through shifts in lattice oxygen and hydroxyl peak intensities. Collectively, these spectroscopic signatures confirm a mechanism adsorption pathway: (i) ion exchange between Ca^2+^ in the alginate network and Cu^2+^, and (ii) chelation via oxygen-rich functional groups.

Figure 7e,f displays the deconvoluted O 1s XPS spectra of Cit-ACP/SA-4 composite beads in pristine and Cu^2+^-loaded states, with quantitative component distributions summarized in Table 6. The O 1s envelope was resolved into three characteristic contributions: lattice oxygen (O^2−^, ~531.2 eV), hydroxyl species (OH^−^, ~532.8 eV), and adsorbed water (H_2_O, ~533.5 eV) [35]. Upon Cu^2+^ uptake, the relative abundance of O^2−^ increased markedly from 33.5% to 53.8%, while adsorbed H_2_O decreased from 24.2% to 15.4% and hydroxyl species declined from 40.7% to 30.4%. This redistribution reflects two concurrent processes: (i) conversion of surface hydroxyl and hydration-layer water into metal–oxygen coordination bonds during Cu^2+^ complexation, and (ii) partial displacement of structural Ca^2+^ by Cu^2+^, generating new Cu–O lattice environments. The concomitant reduction in hydroxyl and hydration signatures, coupled with the rise in metal-coordinated oxygen, provides direct spectroscopic evidence for chelation between Cu^2+^ and oxygen-donor functional groups (carboxylate, phosphate, hydroxyl) inherent to the citrate-stabilized ACP and alginate matrix.

Figure 8a depicts the FTIR spectra of Cit-ACP/SA-4 composite beads before and after Cu^2+^ adsorption. Initially, distinct peaks at 1416 cm^−1^ and 1595 cm^−1^ were observed, corresponding to the symmetric and asymmetric stretching vibrations of carboxyl groups, respectively. Post-adsorption, these peaks shifted to 1412 cm^−1^ and 1586 cm^−1^, indicating coordination interactions between carboxyl groups and Cu^2+^ ions [28]. Additionally, the C–O stretching vibration peak at 1004 cm^−1^ shifted to 1024 cm^−1^ following Cu^2+^ uptake, suggesting that C–O groups also contributed to Cu^2+^ binding [15]. These spectral shifts highlight the role of oxygen-donor ligands in metal ion coordination. Figure 8b illustrates the XRD patterns of Cit-ACP/SA-4 before and after Cu^2+^ adsorption. Prior to adsorption, the pattern displayed a broad diffuse halo within the 2θ range of 20–35°, characteristic of an amorphous structure [19]. After adsorption, two weak diffraction peaks emerged at 26.00° and 32.07°, corresponding to the (002) and (211) planes of hydroxyapatite, as identified by the standard reference card PDF #09-0492 [36]. No other significant diffraction peaks were noted, indicating minimal phase transformation beyond this localized crystallization. This suggests that during the copper removal process, some of the amorphous calcium phosphate may have been converted into a copper-calcium apatite phase.

**Figure 9:**
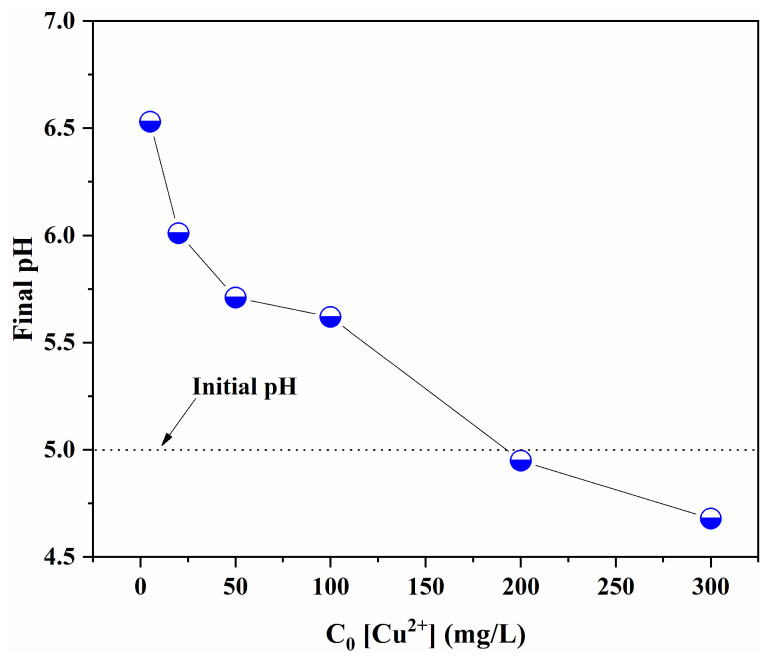
pH variation in the solution after copper adsorption of the sample Cit-ACP/SA-4.

To probe interfacial transformations during Cu^2+^ sequestration, solution pH was monitored before and after adsorption across varying initial Cu^2+^ concentrations (Figure 9), with initial pH fixed at 5.0. At low metal loading (5 mg/L), pH rose markedly to 6.53 post-adsorption. As initial concentration increased to 100 mg/L, equilibrium pH gradually declined to 5.62 yet remained above the initial value. This alkalization stems from Ca^2+^–Cu^2+^ ion exchange: displacement of crosslinking Ca^2+^ in the alginate matrix and structural Ca^2+^ within amorphous calcium phosphate releases OH^−^ equivalents, which is consistent with the diminished Ca 2p XPS signal observed after adsorption.

Conversely, at elevated Cu^2+^ loadings (200–300 mg/L), equilibrium pH fell below the initial value (4.95 → 4.68). This acidification aligns with XRD evidence of nascent crystalline phases (Figure 8b) and is attributed to two concurrent processes: (i) precipitation of Cu-substituted hydroxyapatite, which consumes solution hydroxide ions (OH^−^); and (ii) proton release during Cu^2+^ coordination with surface ≡Ca–OH sites on the evolving copper-calcium phosphate phase [8].

**Figure 10:**
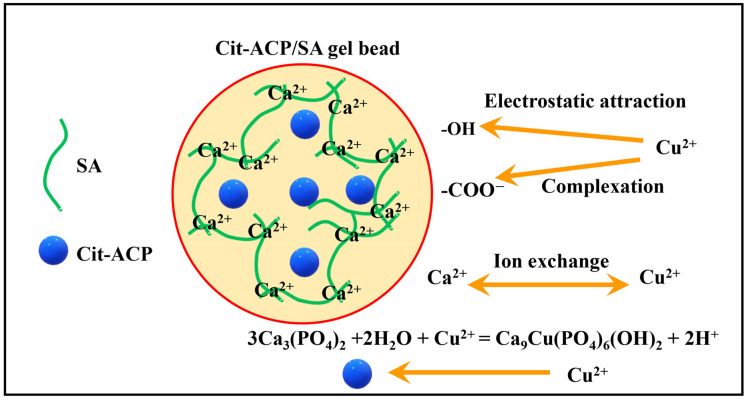
Possible mechanism on absorbing Cu(II) by the sample Cit-ACP/SA-4.

Integrating multi-technique evidence—XPS (Cu 2p emergence, Ca 2p attenuation), FTIR (carboxyl/C–O peak shifts), XRD (phase transformation), and pH trajectory—a dual-stage adsorption mechanism is proposed as shown in Figure 10—(a) ion exchange-dominated stage (low [Cu^2+^] or early adsorption): Cu^2+^ displaces Ca^2+^ from alginate crosslinks and ACP framework, elevating solution pH; (b) surface precipitation/complexation stage (high [Cu^2+^] or late adsorption): following Ca^2+^ depletion, Cu^2+^ reacts with residual phosphate/hydroxyl groups to form copper-calcium apatite and surface complexes, consuming OH^−^ and lowering pH. This mechanistic duality explains the non-monotonic pH response and rationalizes the material’s high capacity.

Compared with other adsorbents, the Cit-ACP/SA gel beads exhibit significant advantages. In particular, Cit-ACP/SA-4 shows a higher adsorption amount than most adsorbents reported in the literature (Table 7). Meanwhile, the adsorbent is facile to prepare, environmentally friendly, and easy to separate after adsorption, highlighting its potential for practical application in the remediation of copper-containing wastewater. Cit-ACP/SA-4 demonstrates superior Cu^2+^ uptake relative to numerous reported adsorbents (Table 6), while offering distinct practical merits: (i) one-pot, aqueous-phase synthesis under ambient conditions; (ii) biodegradable, non-toxic components (alginate, citrate-stabilized ACP); (iii) macroscopic bead morphology enabling rapid solid–liquid separation without filtration aids. Thus, Cit-ACP/SA-4 could be as a scalable, eco-compatible candidate for copper-laden wastewater remediation. For the spent Cit-ACP/SA beads loaded with Cu(II), we envision several practical strategies: (1) copper recovery via acid elution (e.g., with dilute HCl or H_2_SO_4_), though the metastable ACP phase may undergo transformation during this process; (2) direct reuse as a catalyst, as Cu(II)-alginate composites have been reported as active catalysts for organic synthesis; (3) incineration for volume reduction and metal enrichment, with the resulting ash managed as hazardous waste; and (4) secure landfill disposal following characterization to determine its hazard status in compliance with local regulations.

## 4. Conclusions

In conclusion, this study establishes the ambient-condition fabrication of citric acid-stabilized amorphous calcium phosphate/sodium alginate (Cit-ACP/SA) composite beads as a structurally tailored adsorbent for Cu^2+^ remediation. Material characterization confirmed the phase-pure amorphous nature of the incorporated calcium phosphate phase and a texturally favorable specific surface area (90.15 m^2^/g). Under optimized conditions (initial [Cu^2+^] = 300 mg/L), Cit-ACP/SA-4 attained an exceptional equilibrium uptake amount of 296.72 mg/g at 25 °C, 301.63 mg/g at 35 °C and 307.76 mg/g at 45 °C. Adsorption behavior conformed rigorously to the Freundlich isotherm and pseudo-second-order kinetic frameworks, indicating monolayer chemisorption governed by valence interactions. The composite demonstrated remarkable operational resilience across pH 3.0–5.5 and maintained high selectivity despite background electrolytes (Na^+^, K^+^, Mg^2+^, Ca^2+^), with minimal interference except for mild Ca^2+^ competition. Multimodal mechanistic analysis (XPS, FTIR, XRD, pH trajectory) revealed a synergistic adsorption pathway: (i) surface complexation via carboxylate/hydroxyl functional groups of alginate and citrate; (ii) ion exchange between aqueous Cu^2+^ and structural Ca^2+^ within the ACP framework; (iii) in situ transformation of amorphous calcium phosphate into copper-substituted hydroxyapatite, accompanied by surface precipitation phenomena.

While this work demonstrates the excellent Cu(II) adsorption performance of Cit-ACP/SA beads, several aspects warrant further investigation. First, the metastable nature of amorphous calcium phosphate limits the reusability of the adsorbent; future efforts should focus on stabilizing the amorphous phase through compositional or structural modifications. Second, the adsorption behavior under real wastewater conditions (e.g., presence of competing ions, organic matter, and variable pH) needs to be evaluated. Finally, recovering the adsorbed copper as a valuable resource from the spent beads should be explored to align with circular economy principles.

## Figures and Tables

**Table 1:** Sample codes and corresponding mass of SA and Cit-ACP for composite beads with different Cit-ACP contents.

Sample Code	SA (g)	Cit-ACP (g)	CaCl_2_ Crosslinker (wt%)
Cit-ACP/SA-1	1	0	2
Cit-ACP/SA-2	1	0.25	2
Cit-ACP/SA-3	1	0.68	2
Cit-ACP/SA-4	1	1.5	2
Cit-ACP/SA-5	1	4.0	2

**Table 2:** Fitted kinetic parameters for Cu^2+^ adsorption onto Cit-ACP/SA-4 composite beads, derived from pseudo-first-order and pseudo-second-order models.

Samples	Pseudo-First-Order Model			Pseudo-Second-Order Model		
	q_e_ (mg/g)	k_1_ (1/min)	R^2^	q_e_ (mg/g)	k_2_ (g/mg·min)	R^2^
Cit-ACP/SA-4	91.8	0.019	0.991	118.3	0.062	0.999

**Table 3:** Fitted isotherm parameters for sorption onto Cit-ACP/SA-4 composite beads derived from Langmuir and Freundlich model analyses.

Sample	Temp. (°C)	Langmuir Constants				Freundlich Constants		
		*q*_m (cal)_ (mg/g)	*q*_m (exp)_ (mg/g)	*K_L_* (L/mg)	*R^2^*	*K_F_* (mg^1−n^·L^n^/g)	*n*	*R* ^2^
Cit-ACP/SA-4	25	454.55	296.72 ± 12.2	0.028	0.966	14.73	1.33	0.995
	35	460.83	301.63 ± 7.4	0.031	0.969	16.12	1.34	0.996
	45	465.12	307.76 ± 13.6	0.034	0.962	17.82	1.35	0.996

**Table 4:** Derived thermodynamic parameters for Cu^2+^ sorption onto Cit-ACP/SA-4 composite beads across experimental temperatures.

Adsorbent	Temp (K)	Δ*G*° (kJ/mol)	Δ*H*° (kJ/mol)	Δ*S*° (J/mol·K)
Cit-ACP/SA-4	298	−6.18	8.314	48.64
	308	−6.67		
	318	−7.15		

**Table 5:** Parameters of C 1s high-resolution XPS spectra for Cit-ACP/SA-4 composite beads in pristine versus Cu^2+^-exposed states.

Sample	Peak	BE (eV)	Percent (%)
Cit-ACP/SA-4	C-C	284.8	25.2
	C-O	286.7	55.1
	O-C=O	288.5	19.7
Cit-ACP/SA-4-Cu	C-C	284.8	36.2
	C-O	286.5	36.2
	O-C=O	288.3	27.6

**Table 6:** Parameters of O 1s high-resolution XPS spectra for Cit-ACP/SA-4 composite beads in pristine versus Cu^2+^-exposed states.

Sample	Peak	BE (eV)	Percent (%)
Cit-ACP/SA-4	O^2−^	531.2	33.5
	OH^−^	532.8	41.7
	H_2_O	533.5	24.8
Cit-ACP/SA-4-Cu	O^2−^	530.7	53.8
	OH^−^	531.9	30.4
	H_2_O	533.0	15.8

**Table 7:** Comparison of copper ion adsorption properties with reported adsorbents.

Adsorbent	pH	Adsorbent Dosage (g/L)	Initial Concentration (mg/L)	Temperature (°C)	Adsorption (mg/g)	Reference
Cit-ACP/SA-4	5.0	0.8	300	45	307.76	This work
SA/sodium humate@Polyacrylamide	5.0	1	200	40	134.65	[37]
Carboxymethylcellulose-SA hydrogel microspheres	5.0	2	400	25	64.10	[10]
L-arginine Modified Alginate Aerogels	5.0	0.6	200	50	237.45	[35]
Hydroxyapatite modified sludge-based biochar	6.0	1	150	25	89.98	[17]
Magnetic-biochar/alginate beads	5.0	1	200	30	234.10	[38]
GO/MMT/alginate aerogels	6.0	0.2	10	45	50.8	[28]
MXene/polyaniline/SA composite gel	4.0	0.04	1000	45	255.81	[29]
SA/carboxymethylcellulose/Mg(OH)_2_ hydrogel	5.0	1.2	500	25	215.68	[15]
SA/chitosan/montmorillonite-base aerogel	6.0	0.5	400	25	203.99	[39]

Note: the adsorption amount data listed in the table is derived from experimental results reported in the literature.

## Data Availability

The article and raw data include the original contributions made in this study. The data are available from the corresponding author upon reasonable request.

## Supplementary Materials

The following supporting information can be downloaded at: https://www.mdpi.com/article/10.3390/nano16110662/s1. Figure S1: Standard curve showing the linear relationship between copper ion concentration and absorbance (pH = 5.0). The regression equation was Y = 0.17013X - 0.0018 with a correlation coefficient (R_2_) of 0.999. Figure S2: Effect of adsorbent dosage on (a) Cu(II) removal efficiency and (b) equilibrium adsorption amount of Cit-ACP/SA-4 beads. Initial Cu(II) concentration: 200 mg/L; temperature: 25 °C; pH: 5.0; contact time: 24 h.
