# Supplementary material for: Enhanced Removal of Copper Ions from Aqueous Solution by Citrate-Stabilized Amorphous Calcium Phosphate Nanoparticles/Sodium Alginate Composite Hydrogel Beads
Source: Nanomaterials (Basel). 2026 May 24;16(11):662. doi: 10.3390/nano16110662 (PMC13257919; doi:10.3390/nano16110662)
Supplement: Supplementary file 1 [file nanomaterials-16-00662-s001.zip › nanomaterials-4273275-supplementary.pdf]

nanomaterials

Supplementary Materials

Enhanced Removal of Copper Ions from Aqueous Solution by Citrate-Stabilized Amorphous Calcium Phosphate Nanoparticles/Sodium Alginate Composite Hydrogel Beads

Miaomiao Wang, Yuwei Jiang and Junjun Tan *

Hubei Provincial Key Laboratory of Green Materials for Light Industry, School of Material Science and Chemical Engineering, Hubei University of Technology, Wuhan 430068, China

* Correspondence: tanjunjun2011@hbut.edu.cn

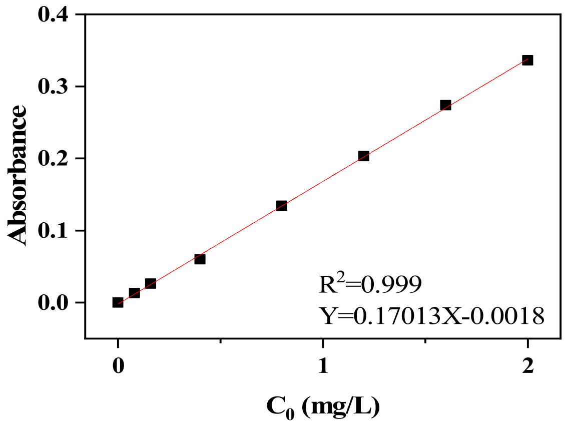

Figure S1. Standard curve showing the linear relationship between copper ion concentration and absorbance (pH = 5.0). The regression equation was Y = 0.17013X - 0.0018 with a correlation coefficient (R^2) of 0.999.

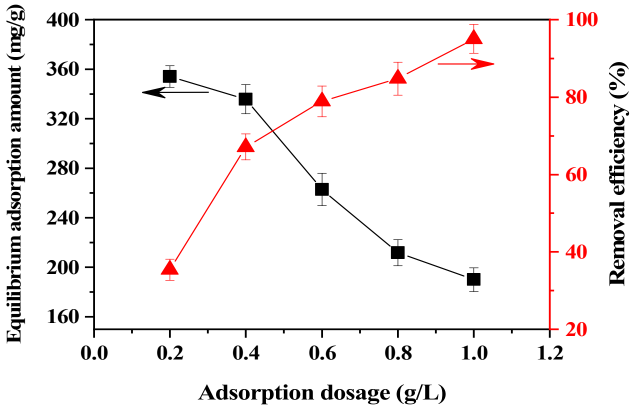

Figure S2. Effect of adsorbent dosage on (a) Cu(II) removal efficiency and (b) equilibrium adsorption number of Cit-ACP/SA-4 beads. Initial Cu(II) concentration: 200 mg/L; temperature: 25 °C; pH: 5.0; contact time: 24 h.
